# Remote Assessment of Video-Recorded Oral Presentations Centered on a Virtual Case-Based Module: A COVID-19 Feasibility Study

**DOI:** 10.7759/cureus.8726

**Published:** 2020-06-20

**Authors:** Conrad Krawiec, Abigail Myers

**Affiliations:** 1 Pediatric Critical Care Medicine, Penn State Health Children's Hospital, Hershey, USA; 2 Pediatrics, Penn State Health Children's Hospital, Hershey, USA

**Keywords:** assessment in health professions education, covid-19, undergraduate medical education

## Abstract

Introduction

The coronavirus disease 2019 (COVID-19) pandemic has resulted in the suspension of our pediatric clerkship, which may result in medical student skill erosion due to lack of patient contact. Our clerkship has developed and assessed the feasibility of implementing a video-recorded oral presentation assignment and formative assessment centered on virtual case-based modules.

Methods

This retrospective study examined the feasibility of providing a remote formative assessment of third-year medical student video-recorded oral presentation submissions centered on virtual case-based modules over a one-week time period after pediatric clerkship suspension (March 16th to 20th, 2020). Descriptive statistics were used to assess the video length and assessment scores of the oral presentations.

Results

Twelve subjects were included in this study. Overall median assessment score [median score, (25th, 75th percentile)] was 5 (4,6), described as “mostly on target” per the patient presentation rating tool.

Conclusion

Patient-related activities during the pediatric clerkship were halted during the COVID-19 pandemic. This study demonstrated the possibility of remotely assessing oral presentation skills centered on virtual case-based modules using a patient presentation tool intended for non-virtual patients. This may prepare students for their clinical experiences when COVID-19 restrictions are lifted. Future studies are needed to determine if suspended clerkships should consider this approach.

## Introduction

In 2020, the coronavirus disease 2019 (COVID-19) pandemic resulted in the unprecedented prolonged closure of educational institutions worldwide to curb the spread of the virus [1,2]. Medical students were included in this group of learners per the guidance of the Association of American Medical Colleges (AAMC) [3]. Thus, our institution temporarily suspended the clinical portion of the pediatric clerkship.

Electronic resources exist to supplement the pediatric clerkship curriculum, thus key aspects can be taught remotely [4-7]. One aspect that electronic sources lack, however, is patient contact. Lack of patient contact results in the inability to practice clinical skills, including interviewing or orally presenting patients recently seen. These clinical skills are often assessed during the pediatric clerkship and students will often specifically receive feedback on these skills [8]. They are also prioritized by some clerkships for the summative evaluation as students must develop these skills to demonstrate they can assess a patient and synthesize their medical knowledge [8].

At our institution, we have instituted a remote learning curriculum for our third-year medical students starting at the end of April 2020. When COVID-19 restrictions are lifted, our students will undergo two weeks of patient contact time. Because our students will not have been in a clinical environment for a prolonged time period, they may have difficulty transitioning [9]. To minimize the impact this transition will have on our students, our pediatric clerkship has developed a video-recorded oral presentation assignment centered on a virtual case-based module with remote formative assessment. Our goal was to enhance the development of this clinical skill remotely thereby allowing students to focus on clinical skill development in areas that cannot be achieved without patient contact (i.e., patient interviewing) when restrictions are lifted.

The objective of this study is to demonstrate the feasibility of student video-recording an oral presentation centered on a virtual case-based module and having our attending faculty members provide a formative assessment. The study hypothesis is that it is feasible to assess and provide formative feedback on video-recorded oral presentations by pediatric attending faculty members using a patient presentation rating tool intended for non-virtual patients.

## Materials and methods

Study design

This is a feasibility study requesting students to video-record an oral presentation centered on a virtual case-based module for formative assessment during a time period (March 16th, 2020 until March 19th, 2020) when Pennsylvania State College of Medicine third-year medical students were abruptly restricted from providing direct patient care during the pediatric clerkship. A retrospective review of faculty submitted formative assessments of the video-recorded oral presentations centered on virtual case-based modules was completed. This study was reviewed by our institution’s review board and determined to be non-human research.

Subject population

Third-year medical students - (1) part of our institution’s traditional curriculum, (2) rotated at the pediatric clerkship’s primary site or off-campus affiliate sites during the first month of the academic year (2020-2021), (3) were abruptly restricted from direct patient care due to the COVID-19 pandemic, and (4) completed a video-recorded oral presentation centered on a virtual case-based module - were included in this study. Students who were part of the longitudinal integrated curriculum were excluded.

Clerkship overview

The pediatric clerkship at our institution is a four-week rotation with the following clinical requirements: outpatient clinic, nursery, and inpatient service. On March 16th, third-year students were restricted from direct patient care, thus only three weeks of the clerkship was completed.

Video-recorded oral presentation assignment and assessment creation

The video-recorded oral presentation assignment was developed by a pediatric clerkship director experienced in inpatient medicine and an outpatient pediatrician. A patient presentation tool developed by Lewin et al. was utilized for this assessment [10].

Using behavioral and verbal anchors, the patient presentation tool assesses various oral presentation sections including patient history, physical exam and diagnostic study results, summary statement, assessment and plan, clinical reasoning/synthesis of information, and general aspects (organization, speaking style) based on a 5-point scale (5 being the highest) [10]. Overall assessment of presentation is based on a 9-point scale (9 being the highest and described as “well above expectations”). Eight faculty members were recruited to use this tool as they were assessing the video recordings.

Video-recorded oral presentation assignment implementation

Starting on March 16th, 2020, the subjects were provided a remote learning curriculum and were notified of the video-recorded oral presentation assignment. They were informed that the pediatric clerkship will be graded pass/fail, that submission of a video-recorded oral presentation for formative assessment will be required, and was due on March 19th, 2020. The subjects were instructed to (1) video-record an oral presentation of either a patient they have seen during the course of the clerkship or after completing a virtual online case-based module through Aquifer © (Lebanon, New Hampshire, USA) and (2) upload the assignment via the Instructure Canvas (Salt Lake City, Utah, USA) learning management system. Students were given specific directions including the use of professional attire, limiting the video-recording to 10 minutes, and requesting students to review the video prior to submission for clarity and organization. After receiving the video-recordings, the files were securely distributed through the CANVAS © learning management system among eight pediatric attending faculty volunteers who reviewed and provided formative assessment scores of the oral presentation.

Data collection

All completed assignments were collected using the Instructure Canvas learning management system. Using the Canvas learning management system, we extracted the following data: overall video-recorded oral presentation rating scores and video-recorded oral presentation scores divided by section as outlined by the patient presentation tool [10].

Virtual case-based module

If students elected to give an oral presentation based on a virtual case-based module, we asked students to complete the pediatric Aquifer © case-based module 3, a 3-year-old male seen for a well-child visit [4]. This case was chosen as it provides a robust history and physical examination, tasks the student to identify and prioritize problems uncovered during this visit, allows the student to apply a differential diagnosis when appropriate, formulate a management plan, and practice their organization skills during the oral presentation.

Statistics

We used descriptive statistics to assess the study population in terms of length of presentation, type of patient presented, and assessment scores based on the patient presentation tool [10]. Formative assessment of each oral presentation was reported in the median and interquartile range.

## Results

Overview

Twelve individual oral presentation videos centered on the virtual case-based module were included in this study. Median video length [median time (mm:ss), (25th, 75th percentile)] was 06:20 (05:04,07:21).

Video-recorded oral presentation assessment scores

Overall, median overall formative assessment score [median score, 25th, 75th percentile] of video-recorded oral presentation centered on virtual case-based modules was 5 (4,6), described as “mostly on target” per the patient presentation tool [10]. The lowest items scored were pertinent positives and negatives of the differential diagnosis [2 (1,3)] (Table 1).

**Table 1:** Median Patient Presentation Rating Tool Section Scores of Video-Recorded Oral Presentations Centered on Virtual Case-Based modules Note: Sections scored on a 1 to 5 scale, 5 being the highest score Patient Presentation Rating Tool for Oral Case Presentations [10].

Patient Presentation Rating Tool Section Scores	Median Score (25^th^, 75^th^)
1. Chief complaint noted either before history of present illness (HPI) or as part of introductory sentence	4 (3,5)
2. HPI starts with clear patient introduction including patient's age, sex, pertinent active medical problems and reasons for admission	4.5 (3,5)
3. HPI is organized so that chronology of important events is clear	3 (2,4)
4. The past medical history, family history, social history, and review of systems include only elements related to active medical problems	3 (3,3)
5. Begins with a general statement	4 (3, 4.25)
6. Presents all vital signs (and growth parameters if patient is a child)	3.5 (3,5)
7. Includes a targeted physical exam stating the positive and negative findings that distinguish the diagnoses under consideration and any other abnormal findings	4 (3,4.75)
8. Organizes laboratory data and results of other diagnostic tests to distinguish between possible diagnoses	4 (3.5, 4.5)
9. Begins assessment with a summary statement that synthesizes the critical elements of the patient's history, physical exam and diagnostic studies into one sentence	3 (2,4)
10. Includes a prioritized problem list (by systems only if appropriate) including all active problems	4 (3,5)
11. Provides an appropriate differential diagnosis for each problem	2 (1,3)
12. States the diagnostic/therapeutic plan that targets each problem; each item in the plan relates to something listed on the problem list	3.5 (3,4.25)
13. The presentation included the pertinent positives and negatives from the history and physical to support the differential diagnosis and plan	3 (2,4)
14. At the end of the presentation I had a clear picture of this patient's situation and what needed to be done next	3 (3,4)
15. Overall organization	3 (3,3.25)
16. Speaking style	5 (3.75, 5)

## Discussion

Oral presentations are an essential clinical skill that facilitates physician to physician communication, improves efficiency on rounds, and enables individual as well as group learning [8]. It also can be complex and time-consuming as students must use their medical knowledge and clinical reasoning skills to select the pertinent details to present from a patient’s history, physical, diagnostic, and laboratory tests [8,10]. In this study, we hypothesized that video-recorded oral presentations centered on a virtual case-based module can undergo a formative assessment. This study successfully demonstrated that a formative assessment can be remotely provided for video-recorded presentations based on virtual case-based modules. These results imply that this form of assessment is possible, may prepare students for the eventual live clinical experience (with patient contact), and potentially optimize the transition period from COVID-19 remote learning to a post-COVID-19 clinical patient experience.

To our knowledge, a pediatric clerkship has never been halted in this manner for a prolonged period due to a nationwide health emergency. Because of this, our pediatric clerkship, like others across the United States was placed in an unprecedented situation, potentially placing our students at risk of achieving suboptimal competency in various clinical areas [11]. Novel approaches are necessary to ensure that our students, who were hastily restricted during their pediatric clerkship and future students that have yet to complete their pediatric clerkship, are adequately trained [11].

Our institution’s current plans are for each clerkship to institute a remote learning curriculum and complete a two-week immersive clinical experience in each of the core clerkships. The remote learning curriculum will allow students to learn the basic concepts relevant for pediatrics and the two-week patient contact experience will allow students to apply their knowledge. When the two-week patient contact experience begins, however, the transition period may be difficult. Students will not have seen a patient (possibly for months) and similar to transitioning from the pre-clerkship to clerkship years, students may be overwhelmed by clerkship logistics, expectations, and adjusting to the clinical culture [12]. In all, students may be overwhelmed by this and the number of tasks they must complete in a short time period post-clerkship suspension, potentially limiting their clinical experience.

Thus, it is the clerkship’s responsibility to ensure that students in a remote curriculum continue to be comparably trained and are provided as many similar clinical experiences as possible to ease the transition that will occur on clerkship reinstatement. While the pediatric clerkship is currently limited in allowing students to see patients during the remote learning experience, there are other ways that students can be robustly prepared for the clinical environment. The area that our clerkship elected to focus on is the oral presentation.

If students are rigorously prepared to practice oral presentation skills using pediatric faculty members (that they will eventually present to), students may start to apply their communication, medical knowledge, and clinical reasoning skills earlier and potentially focus their clinical skills on other areas that they cannot easily achieve remotely (i.e., history taking and physical examination and providing live patient care) when they return to clerkship. Students may also have a better understanding of their expectations, roles, and responsibilities of this skill for our clerkship and thus are better prepared to provide meaningful patient care and be effective team members sooner.

In our study, we found that it is feasible for students to submit a video-recorded oral presentation centered on a virtual case-based module and recruit pediatric attending faculty members to assess and provide formative feedback. We also found that the overall median scores were “mostly on target” according to the patient presentation tool. The students who completed these assessments were the first students for the academic year, thus these results may indicate that these students developmentally require more practice. Alternatively, these results may indicate that because these assessments are formative and the clerkship is now pass/fail, these students were given the feedback necessary to improve their skills. Finally, students may not have received enough individual educational attention during the normal clinical workflow and thus were not given enough instruction. More studies are necessary to determine if these assessments are consistent.

Limitations

There were several limitations to this study. This includes its small sample size, the short intervention period, and the lack of randomization. The patient presentation rating tool intended for live patients was used without the opportunity to validate it for use in virtual case-based modules due to the haste in its implementation. Future studies will be required to validate the tool for this purpose. Student perception is also unknown regarding the effectiveness of this assessment technique, thus future qualitative studies are planned to determine this.

## Conclusions

Our pediatric clerkship was suddenly curtailed during the COVID-19 pandemic. The students were provided a remote learning curriculum to emphasize pediatric concepts but may not be able to demonstrate their clinical skills in communication, data synthesis, and patient assessment. Our study demonstrated that it is possible to assess oral presentation skills centered on virtual case-based modules using a patient presentation rating tool intended for non-virtual patients and may potentially prepare students for their clinical experiences when COVID-19 restrictions are lifted. Future studies are needed to determine if suspended clerkships should consider this approach.
